# Hypothalamic atrophy in primary lateral sclerosis, assessed by convolutional neural network-based automatic segmentation

**DOI:** 10.1038/s41598-025-85786-6

**Published:** 2025-01-10

**Authors:** Jan Kassubek, Francesco Roselli, Simon Witzel, Johannes Dorst, Albert C. Ludolph, Volker Rasche, Ina Vernikouskaya, Hans-Peter Müller

**Affiliations:** 1 Dept. of Neurology, University of Ulm, Oberer Eselsberg 45, 89081 Ulm, Germany; 2 German Center for Neurodegenerative Diseases (DZNE), Ulm, Germany; 3 Department of Internal Medicine II, Ulm University Medical Center, Ulm, Germany; 4 Core Facility Small Animal MRI, University of Ulm, Ulm, Germany

**Keywords:** Hypothalamus, Neuronal Networks, Metabolism, Magnetic Resonance Imaging, Volumetry, Amyotrophic Lateral Sclerosis, Primary Lateral Sclerosis, Amyotrophic lateral sclerosis, Amyotrophic lateral sclerosis

## Abstract

Primary lateral sclerosis (PLS) is a motor neuron disease (MND) which mainly affects upper motor neurons. Within the MND spectrum, PLS is much more slowly progressive than amyotrophic laterals sclerosis (ALS). `Classical` ALS is characterized by catabolism and abnormal energy metabolism preceding onset of motor symptoms, and previous studies indicated that the disease progression of ALS involves hypothalamic atrophy. Very limited weight loss is observed in patients with PLS, which raises the question of whether there are also less hypothalamic alterations. The purpose of this study was to quantitatively investigate the hypothalamic volume in a group of PLS patients and to compare it with ALS and controls. Recently, we have introduced automatic hypothalamic quantification method based on the use of convolutional neural network (CNN) to reduce human variability and enhance analysis robustness. This CNN of U-Net architecture was applied for automatic segmentation of the hypothalamus and intracranial volume (ICV) to allow adjustments of the hypothalamic volume between subjects with different head sizes respectively. Automatic segmentation and volumetric analysis were performed in high resolution T1 weighted MRI volumes (acquired on a 1.5 T MRI scanner) of 46 PLS patients in comparison to 107 healthy controls and 411 `classical` ALS patients, respectively. Significant hypothalamic volume reduction was observed in PLS (818 ± 73 mm^3^) when compared to controls (852 ± 77 mm^3^); significant hypothalamic volume reduction was also confirmed in ALS (823 ± 84 mm^3^), in support of previous studies. No significant differences were found in normalized hypothalamic volumes between ALS patients and PLS patients at the group level. This unbiased CNN-based hypothalamus volume quantification study demonstrated similarly reduced hypothalamus volume in PLS and ALS patients, despite the clinical phenotypic differences.

## Introduction

Within the spectrum of adult motor neuron diseases (MND), primary lateral sclerosis (PLS) is a characteristically slowly progressive and selective neurodegenerative disorder primarily affecting the upper motor neuron (UMN)^1^. As a clinically defined syndrome, the current core consensus diagnostic criteria include progressive UMN dysfunction in at least two of three regions (lower extremity, upper extremity, bulbar) for at least 2 years, in the absence of active lower motor neuron (LMN) degeneration or sensory symptoms or alternative explanation^1,2^. Despite this separate clinical definition and the much slower clinical course, PLS has still to be viewed in the context of the most common MND amyotrophic lateral sclerosis (ALS) with its combined UMN and LMN degeneration and generally rapidly fatal course. Here, PLS has been considered to be a phenotypical variant of ALS^3^. In support, for example, MRI studies have demonstrated the same microstructural corticoefferent affectation patterns in PLS patients as in ALS^4,5^.

ALS is traditionally conceptualized as a neurodegenerative multisystem disease with a dominant motor phenotype resulting from the affection of primarily the motor neurons—within the non-motor symptoms of ALS with substantial impact on patient well-being and overall survival, there is a substantial hypermetabolic phenotype which accompanies the clinical onset of ALS^6^. Studies of the hypothalamus (as one key brain structure involved in body composition^7^) by volume analysis with manual delineation in T1-w high-resolution MRI scans had shown that the hypothalamus is significantly atrophied in ALS^8^ as well as in ALS-FTD patients^9^. These results have been confirmed by different methodological approaches in different ALS cohorts^10,11^ with the exception of smaller cohorts^12^, and have been correlated to patients` survival^13^. Only limited weight loss is observed in patients with PLS^14^, what raises the question if the imaging phenotype of PLS also shares hypothalamic atrophy with ALS. Therefore, we quantified the hypothalamic volumes in a group of PLS patients vs. healthy controls and vs. `classical` ALS by a novel automatic approach with the use of convolutional neural networks (CNN)^15^.

## Results

**Fig. 1:**
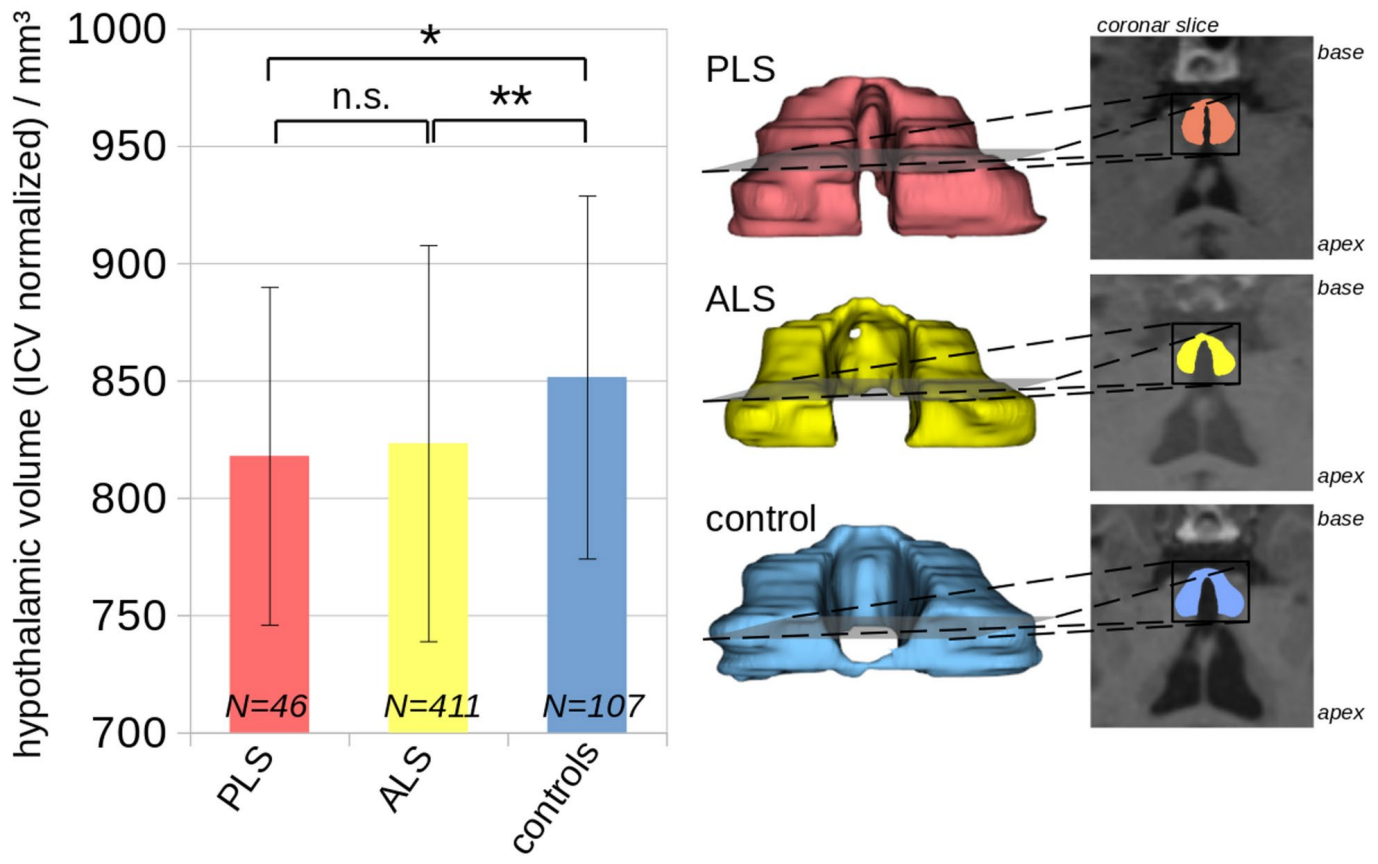
**Left:** ICV normalized hypothalamic volumes at the group level. **Right:** Examples of hypothalamus segmentation for a control, an ALS patient and a PLS patient. n.s.—not significant.

The hypothalamus volume analysis in the group comparison showed a significant reduction in hypothalamic volume in the PLS group (818 ± 73 mm^3^) compared to controls (852 ± 77 mm^3^) (-3% vs. controls, p = 0.013)^15^. When subdividing the PLS group into probable and definite PLS, the comparison of the hypothalamic analysis between both groups showed no differences – probable PLS group (N = 28; 818 ± 63 mm^3^), definite PLS group (N = 18; 818 ± 89 mm^3^). The ALS group (823 ± 84 mm^3^) showed no differences to the PLS group, but also significant differences to the controls (-3% vs. controls, p = 0.002) (Fig. 1).

The resulting hypothalamic volumes showed neither sex- nor age-dependence; therefore, no correction with these covariates has been performed.

The association analysis of the hypothalamic volumes of PLS patients revealed no significant correlation neither to disease duration (C = 0.03) nor to disease progression (slope of ALS-FRS-R, C = -0.14) (Table 1).

**Table 1:** Subject characteristics.

	Sex (m/f)	Age mean ± std median (range)	Slope*	ALS-FRS-R	Disease duration / years
All PLS (N = 46)	26 / 20	59.8 ± 10.7 59 (35–86)	2.3 ± 1.4	39 ± 7	4.2 ± 4.9
Probable PLS (N = 28)	17 / 11	59.9 ± 11.2 57 (39–86)	2.9 ± 0.9	42 ± 2	1.6 ± 1.0
Definite PLS (N = 18)	9 / 9	59.7 ± 10.1 60 (35–74)	1.9 ± 1.6	36 ± 8	7.9 ± 5.9
ALS (N = 411)	241 / 170	62.4 ± 11.6 64 (21–93)	5.0 ± 5.3	39 ± 8	1.5 ± 1.4
CON (N = 107)	53 / 54	61.4 ± 10.4 64 (38–82)		-	-
p-value	-	0.8	< 0.001^#^	0.8^#^	< 0.001^#^

ALS-FRS-R – revised ALS functional rating scale^25^. p-value was calculated by Kruskal–Wallis-test; *slope = (48-ALSFRS-R)/(disease duration); #(ALS vs PLS).

## Discussion

This CNN-based analysis of the hypothalamus volume in PLS demonstrated a significant hypothalamic atrophy in comparison to healthy controls which was not significantly different from a disease control group of `classical` ALS patients. The finding of reduced hypothalamic volumes in ‘classical’ ALS was confirmed as a control investigation by an unbiased automatic analysis approach in a large group of ALS patients; a more detailed analysis, pointing ALS-associated atrophy into the anterior superior subregion of the hypothalamus, was recently performed in a large group of 564 ALS patients by Michielsen and colleagues^16^. These results are in further support of the identical advanced MRI signatures of the MND spectrum diseases ALS and PLS, in contrast to the divergent MRI signature of genetic ALS cases^17^. Hypothalamic atrophy might thus be included in future MRI-based scores which have recently been developed for DTI and texture MRI data in PLS^2^. On the other hand, this finding is at odds with hypothesis that the much less prominent hypermetabolic state in PLS might have a morphological correlate of an unaltered or less altered hypothalamic volume. In other words, the upper motor neuron phenotype in PLS and the `classical` upper and lower motor neuron phenotype in ALS patients share identical cerebral in vivo presentation of both corticoefferent pathology including the corticospinal tracts and other tract systems^5^ and reduced hypothalamic volume. Therefore, the current neuroimaging study on hypothalamus volume demonstrates another common feature of PLS and ALS supporting that PLS might be a subtype of ALS rather than an independent disease entity – which is the topic of an ongoing clinical discussion^18^. This finding is in line with previous neuropathological studies which have shown TDP-43 pathology in PLS indicating that PLS and ALS are closely related conditions^19,20^. Despite these similarities, PLS is a distinct form of MND with extremely minor involvement of LMN and with much longer survival times and very long disease duration in some cases.

Given the core role of the hypothalamic pathways in metabolic control and its functional changes in diseases like ALS^6^, these findings have to be seen in a context with the changes of the body mass index and with the ALS-associated changes in the composition of visceral and subcutaneous body fat^21^. In that context, it has to be held that the current study was not without limitations. In general, this morphological/volumetric MRI study could not contribute to the understanding of the functional role of the hypothalamus in MND per se. More specifically, we could not perform correlational analyses between hypothalamic volumes and clinical measures like body mass index because these data were not available in the majority of patients. In future prospective studies, brain imaging with hypothalamus volumetry in PLS should be combined with measures of body composition including body mass index or MRI-based indicators like bodyfat compartment determination^22^. As a further limitation, a differentiation with respect to the temporal course between PLS and ALS was not possible on the basis of the cross-sectional study design. The analysis of 1.5T data with an isotropic voxel size of 1.0 mm is appropriate, as these images are included as standard in MRI of neurodegenerative diseases. The hypothalamic volume therefore has an extension of about 8–12 voxels in each spatial direction; this causes partial volume effects, which cannot be neglected. Upsampling to a 0.5 mm grid primarily facilitates the manual determination of ground truth and the visual inspection of the results; whether upsampling improves the quality of the analysis results with regard to partial volume effects is rather uncertain. In any case, the results of the hypothalamic volume reduction should be interpreted at the group level; an application at the individual level would primarily require an increase in the isotropic resolution of the MR images.

The aim of this study was to specifically address a potential regional hypothalamic atrophy in PLS. The additional assessment of volumetric alterations in PLS may provide further information of a global or localized cortical or subcortical atrophy, including techniques like volumetric or cortical thickness measurements, but was beyond the scope of the present study.

In summary, the clinical differences between ALS and PLS might be explained by a higher vulnerability of the second motoneuron in ALS patients which cannot be addressed by brain imaging. The finding of hypothalamic atrophy (of the same magnitude as in ALS) also in PLS might indicate that the protection of the LMN could be associated with some protective factors for metabolic changes which might be a contributor to the much longer survival times in PLS. In that context of potential therapeutic concepts in MND which address metabolic/nutritional factors^23^, The complex interplay between UMN/LMN degeneration and metabolism in different MND will be the topic of future research.

## Material and methods

### Ethical approval

The study has been approved by the Ethics Committee of the University of Ulm (references #19/12 and #20/12) in accordance with the ethical standards laid down in the 1964 Declaration of Helsinki and its later amendments. Written informed consent was obtained from all individual participants included in the study.

### MRI recording

T1-weighted whole head MRI datasets were acquired on a 1.5 T MRI scanner (Symphony, Siemens Medical, Erlangen, Germany). Morphological data were obtained with a MPRAGE sequence (144 sagittal slices, no gap, 1.0 × 1.2 × 1.0 mm^3^ voxels, 256 × 192 × 256 matrix, TE = 4.2 ms, TR = 1600 ms), which is part of a standard clinical MRI examination protocol for patients with motor neuron diseases (MND). Forty-six PLS patients according to the current diagnostic criteria^1^ were investigated; the data collection of the PLS group was performed by a combination of data-base entry and retrospective review of patients’ baseline and follow-up records. Eligible patients had a diagnosis of PLS or suspected PLS at the current evaluation in the centre, as determined by the treating physicians, by application of the current consensus diagnostic criteria^1,2^. The body-mass-index (BMI) in the PLS patients at the time of MRI scanning had an average value of 25 kg/m^2^, the BMI was within the range of normal subjects and therefore gave no indication for hypermetabolism.

One-hundred-and-seven healthy subjects (from the data archive of the Dept. of Neurology, University of Ulm, scanned according to approval by the Ethics Committee of the University of Ulm, references #19/12 and #20/12) without any neurological/psychiatric disease or other medical condition composed the control group; four-hundred-and-eleven patients with sporadic ALS were recruited in the outpatient and inpatient settings of the Department of Neurology, University of Ulm, Germany and composed the ALS group.

Disease progression rate as ALSFRS-R slope (slope = (48-ALSFRS-R)/(disease duration)) was included in the Table 1. *C9orf72* status was negative in all PLS patients; out of the 411 ALS patients, 31 were tested positive on *C9orf72*. No mutations of major genes related to hereditary spastic paraparesis were found in all patients. The Edinburgh Cognitive and Behavioural ALS Screen (ECAS) total score^24^ was applied, and about 40% of the patients demonstrated cognitive impairments in at least one cognitive domain.

### Data preprocessing

**T**1-weighted MRI data were pre-processed using the *Tensor Imaging and Fiber Tracking* (*TIFT*) software package expanded by a volumetric extension package^26^. First, the rigid body normalization of T1-weighted MRI data was performed along the anterior commissure (AC) – posterior commissure (PC) axis such that the coronal cutting plane was perpendicular with respect to the AC-PC axis to correct for individual tilt of the head and to minimize potential partial volume effects. Then, spatial upsampling was performed to improve the accuracy in identifying hypothalamic borders. The hypothalamic section of each dataset was pre-selected in 50 slices of 0.5 mm thickness (matrix 512 × 512, resolution 0.125 × 0.125 mm^2^).

### Data analysis

In a previous study^15^, aiming at enhancing the accuracy and reliability of hypothalamic volume measurements in patients with neurodegenerative diseases, we included a total of 684 T1-weighted whole head MRI datasets. Out of these, 120 datasets (78 ALS patients and 42 healthy controls) obtained a corresponding ground truth segmentation of the hypothalamus^8^. These were used for training and validating of the automatic segmentation approach, which employed a convolutional neural network (CNN) based on U-Net architecture^15^. Additionally, a second network for the automatic segmentation of the intracranial volume (ICV) was implemented. This step was crucial for adjusting hypothalamic volume measurements between subjects with different head sizes, ensuring accurate comparisons. For the final volumetric analysis, 46 PLS patients, 107 controls, and 411 ALS patients, without corresponding ground truth data were included. Details were described in^15^, examples of the segmented hypothalamus are shown in Fig. 1.

Volumetric analysis was performed by calculating the normalized hypothalamus volume as:

1 $${{V}_{hypoth{al}_{norm}}=V}_{hypothal}/{V}_{ICV}*{V}_{{ICV}_{mean}\left(control\right),}$$

where $V$ denotes the automatically segmented volume of individual hypothalamus or ICV, respectively, and ${V}_{{ICV}_{mean}\left(control\right)}$ is the average ICV volume of controls. The multiplication by the mean intracranial volume of the control group was performed to rescale the hypothalamic volume; here, we followed the procedures of previous volumetric analyses (e.g.^27^.

### Statistical analysis

Since manual delineations were not available for the entire dataset, a quality check of the automated segmentation was performed based on the outliers detected in the predicted hypothalamus volume and ICV. Outliers were identified using the interquartile range (IQR) method, where any value falling outside the range [Q1-1.5*IQR; Q3 + 1.5*IQR] was classified as an outlier. These detected outliers (5 controls, 21 ALS, 1 PLS, i.e. less than 5% in each group) were excluded from the calculation of the average normalized hypothalamic volume to ensure the robustness and accuracy of the results.

The differences between ICV normalized hypothalamus volumes in the control and the ALS group were assessed applying unpaired t-test after testing for normality by Shapiro–Wilk test; since normal distributions were the case in all tests, the application of a non-parametric test was not necessary. A *p*-value < 0.05 was assumed statistically significant.

Association analyses between hypothalamic atrophy and clinical parameters were performed by Pearson correlation (after testing for normal distribution).

## Data Availability

The original contributions presented in the study are included in the article, further inquiries can be directed to the corresponding author on reasonable request.
